# Categorical Perception of Mandarin Pitch Directions by Cantonese-Speaking Musicians and Non-musicians

**DOI:** 10.3389/fpsyg.2021.713949

**Published:** 2021-10-14

**Authors:** Si Chen, Yike Yang, Ratree Wayland

**Affiliations:** ^1^Department of Chinese and Bilingual Studies, The Hong Kong Polytechnic University, Hong Kong, SAR China; ^2^Hong Kong Polytechnic University-Peking University Research Centre on Chinese Linguistics, Hong Kong, SAR China; ^3^Department of Linguistics, University of Florida, Gainesville, FL, United States

**Keywords:** categorical perception, musical experience, intrinsic *F*_0_, stimulus duration, tone

## Abstract

**Purpose:** This study is to investigate whether Cantonese-speaking musicians may show stronger CP than Cantonese-speaking non-musicians in perceiving pitch directions generated based on Mandarin tones. It also aims to examine whether musicians may be more effective in processing stimuli and more sensitive to subtle differences caused by vowel quality.

**Methods:** Cantonese-speaking musicians and non-musicians performed a categorical identification and a discrimination task on rising and falling continua of fundamental frequency generated based on Mandarin level, rising and falling tones on two vowels with nine duration values.

**Results:** Cantonese-speaking musicians exhibited a stronger categorical perception (CP) of pitch contours than non-musicians based on the identification and discrimination tasks. Compared to non-musicians, musicians were also more sensitive to the change of stimulus duration and to the intrinsic *F*_0_ in pitch perception in pitch processing.

**Conclusion:** The CP was strengthened due to musical experience and musicians benefited more from increased stimulus duration and were more efficient in pitch processing. Musicians might be able to better use the extra time to form an auditory representation with more acoustic details. Even with more efficiency in pitch processing, musicians' ability to detect subtle pitch changes caused by intrinsic *F*_0_ was not undermined, which is likely due to their superior ability to process temporal information. These results thus suggest musicians may have a great advantage in learning tones of a second language.

## Introduction

Pitch, the perceptual correlate of fundamental frequency (*F*_0_), plays an important role in both music and language. Musicians and tone language speakers have in common enhanced sensitivity to small pitch changes associated with meaningful units, namely melodies for musicians and words for speakers of a tone language. While the effects of musicianship and experience with lexical tones have been separately studied among native and non-native speakers of tone languages, respectively, the effects of musical experience on lexical tone perception among speakers of lexical tone languages have been rarely investigated. To fill this research gap, the current study investigates the effects of musical training and categorical perception (CP) of tones among tonal speakers. Potential factors such as stimulus duration and vowel quality are also explored.

This study aims to better understand the relationship between musical experience and linguistic processing by comparing native Cantonese musicians and non-musicians on their categorization of falling and rising pitch continua, representative of Mandarin tones. The continua are realized on a high [i] and a low vowel [a], associated with a high and a low intrinsic *F*_0_, respectively (Whalen and Levitt, 1995). Unlike Mandarin, Cantonese contrasts six lexical tone categories in open syllables (Xu and Mok, 2012). In addition, while the four Mandarin tones contrast with each other in terms of direction of *F*_0_ movement (level, falling, and rising), three of the six tones in Cantonese tones differ in pitch height. Therefore, Cantonese listeners place more emphasis on average height of *F*_0_ contour than do Mandarin listeners when processing tones (Peng et al., 2012). This may explain why, besides Mandarin tone 2 (high-rising) vs. tone 3 (low-falling-rising), Cantonese speakers have additional difficulties differentiating Mandarin tone 1 (high-level) form Mandarin tone 4 (high-falling) (Hao, 2012). However, due to their denser tonal system, native Cantonese listeners may be more efficient in processing pitch variation than native Mandarin listeners (Lee et al., 1996; Zheng et al., 2012).

### Relationship Between Music and Language

Previous behavioral research has found that musical experience improves lexical tone perception among non-native tone speakers. For example, English-speaking musicians were significantly more skilled at identifying or discriminating Mandarin tones (Alexander et al., 2005; Lee and Hung, 2008) than English-speaking non-musicians, even when only partial *F*_0_ information was present (Lee and Hung, 2008). Additionally, novice English musicians were more accurate and faster than non-musicians in their discrimination of Thai tones (Burnham et al., 2015).

Neurophysiological data has also revealed the facilitative effects of musicality on lexical tone processing. For instance, enhanced event-related brain potentials (ERPs) associated with Mandarin tone deviants were observed in French-speaking musicians compared to non-musicians (Marie et al., 2012). Furthermore, correlations between *F*_0_ tracking quality and the amount of musical training and performance on identification and discrimination of Mandarin syllables were positive (Wong et al., 2007). Similarly, brainstem frequency following response (FFR) to homologs of musical intervals and of lexical tones showed that pitch tracking and pitch strength were more robust for English musicians compared to English non-musicians (Bidelman et al., 2011). The above findings indicated the overlap in music and language perception, suggestive of a common perceptual substrate for the two domains (Maggu et al., 2018). To account for how musical experience may benefit speech processing, Patel (2011, 2012), and Patel (2014) proposed a neurocognitive model, OPERA, which stands for Overlap, Precision, Emotion, Repetition, and Attention. According to this model, speech processing benefits in musicians are attributed to overlapping brain networks engaged during speech and music perception.

In comparison to studies conducted on non-tone speaking musicians and non-musicians (Gottfried and Riester, 2000; Gottfried et al., 2004; Alexander et al., 2005; Gottfried, 2007; Wong and Perrachione, 2007; Lee and Hung, 2008) which largely revealed an overlap between music and language perception, the interaction between music and language among native tone speakers is not conclusive. For example, Tang et al. (2016) found increased neural activity in the discrimination of Mandarin tones (Tone 1 and Tone 2) and musical notes (C4 and G3 in piano timbre) among Mandarin-speaking musicians compared to Mandarin-speaking non-musicians. Similarly, Ong et al. (2020) found that Cantonese musicians were more accurate than non-musicians in their ability to discriminate and identify the most challenging tone pair (T23–T25). On the other hand, Thai musicians in Cooper and Wang (2012) were not superior to Thai non-musicians (or English-speaking musicians) in their ability to associate a change in pitch to a change in lexical meaning. Mok and Zuo (2012) found that, while French and English-speaking musicians outperformed non-musicians in their ability to identify Cantonese tones and their pure tone analogs, such musical advantage was not observed among native Cantonese listeners, suggesting that the linguistic and musical processing may belong to separate but overlapping domains, at least among native tone speakers.

In sum, behavioral and electrophysical studies have found that musical experience facilitates pitch perception among non-tone musicians. However, the facilitative effects of musical experience on pitch perception among native speakers of lexical tones remain inconclusive.

### Musical Experience and Categorical Perception of Lexical Tones

Categorical perception, a classic paradigm in speech perception, refers to the ability to perceive and group linguistically distinct categories with equal physical changes along a continuum (Liberman et al., 1957). The results of prior research suggested that tonal continua were largely perceived categorically by native tone listeners (Abramson, 1975; Wang, 1976; Francis et al., 2003), but continuously by non-native tone listeners (e.g., Hallé et al., 2004; Peng et al., 2010).

It has been reported that musical experience modulates CP of non-speech and speech *F*_0_ continua (Zatorre and Halpern, 1979; Howard et al., 1992; Wu et al., 2015; Zhao and Kuhl, 2015a; Chen et al., 2020), suggesting that experience with the distinguishing of pitch categories defined along an *F*_0_ continuum in the musical domain (i.e., musical notes) may induce CP of both linguistic and non-linguistic *F*_0_ continua. For example, Zatorre and Halpern (1979) found that perception of a continuum of major and minor thirds whose component tones were sounded simultaneously was more categorical among musicians than among non-musicians. Similarly, Howard et al. (1992) found that the ability to label members of a computer-synthesized continuum of major to minor was more categorical among the most musical compared to the least and the moderate musical listeners. Wu et al. (2015) compared identification and discrimination of Mandarin Tone 1–Tone 4 continuum by Mandarin musicians and non-musicians. While the steepness and the location of the category boundary as well as the between-category discrimination were comparable between the two groups, within-category discrimination was found to be enhanced among the musicians. The results suggested that musicality refines low-level auditory perception without interfering with higher-level, categorical processing of lexical tonal contrasts in native tonal listeners. Recently, Chen et al. (2020) found that perception of level-to-rising and level-to-falling pitch continua was more categorical among English-speaking musicians than among English-speaking non-musicians. Zhu et al. (2021) examined CP of Mandarin Tone 2–Tone 4 continuum and its non-speech, pure tone analogs, and found that mean amplitude of the mismatch negativities (MMNs) elicited by within-category deviants was significantly larger among amateur musicians than non-musicians for both types of continua. This result suggests that musical advantage extends to auditory processing of pitch at the pre-attentive level and is not confined to professional musicians. According to Bidelman (2017), musical training may improve the abilities of representing auditory objects and matching incoming sounds to memory templates, and these improved abilities may provide musicians with advantages in CP of speech.

However, musical training does not always promote or enhance CP among either non-native or native speakers of lexical tones. For example, Zhao and Kuhl (2015a) compared the perception of Mandarin Tone 2-Tone 3 continuum among English-speaking musicians, English-speaking non-musicians, and Mandarin-speaking non-musicians and found that in contrast to native Mandarin non-musicians, English-speaking musicians and non-musicians perceived the continuum non-categorically, and while short-term perceptual training improved perception, no evidence of categorical formation among either musicians or non-musicians post training. According to Bidelman et al. (2011), “Pitch encoding from one domain of expertise may transfer to another as long as the latter exhibits acoustic features overlapping those with which individuals have been exposed to from long-term experience or training” (p. 432). Zhao and Kuhl (2015b) compared musical pitch and lexical tone discrimination among Mandarin musicians, Mandarin non-musicians, and English musicians. No difference between Mandarin musicians and non-musicians was found in their sensitivity to lexical tones or in the pattern of within-pair sensitivity to the tone pairs. The English musicians showed significantly higher overall sensitivity to lexical tones than the two Mandarin groups and exhibited a different pattern of within-pair sensitivity, indicating that the processing of musical pitch and lexical pitch might be independent in nature. In addition, Chen et al. (2020) reported that Mandarin musicians did not consistently perceive rising and falling pitch directions more categorically than Mandarin non-musicians. A plausible explanation is that perception of music and speech may implicate distinct processing mechanisms, and the processing of lexical tones makes use of other phonetic cues (e.g., duration and amplitude) besides *F*_0_ (Liu and Samuel, 2004; Lee and Lee, 2010). Maggu et al. (2018)'s finding that Cantonese musicians did not differ from non-musicians on the brainstem encoding of lexical tones, but that they showed a more robust brainstem encoding of musical pitch as compared to non-musicians lends further support to the hypothesis that distinct mechanisms are engaged in the encoding of linguistic and musical pitch among native tone speakers.

To further probe the interaction between musical and linguistic training on pitch perception, we compared CP of Mandarin tones among Cantonese musicians and non-musicians.

### The Role of Stimuli Duration and Vowel Quality in Perception of Tones

It has been found that perception of shorter vowels is more categorical than perception of longer vowels, suggesting the role of stimulus duration in CP. Duration is purported as one possible acoustic dimension affecting representation strength. According to the cue-duration hypothesis, acoustic information of consonants (e.g., formant transitions) is relatively short and less represented while information of formants in vowels is longer and better represented in auditory memory (Fujisaki and Kawashima, 1970). Moreover, the interaction between tones and the perceived vowel duration has been reported in several studies (Yu et al., 2014; Wang et al., 2017). For example, Yu et al. (2014) argues that perceived duration may be affected by *F*_0_ slope and height. Usually, syllables with dynamic *F*_0_ tend to be perceived as longer than those with flat *F*_0_. These results suggest an interaction between perceived duration and tonal shapes. Duration of stimulus also plays a critical role in pitch contour perception. It has been reported that for both native Mandarin and English speakers, the strength of CP increased as stimulus duration increased. In addition, native Chinese listeners showed stronger effects from stimulus duration in terms of category boundary sharpness, between- and within- category discrimination, and peakedness compared to native English listeners (Chen et al., 2017). These results are inconsistent with the cue-duration hypothesis stating that longer stimuli will be processed with weaker CP. They also revealed an influence of tone language background to the duration effect (Chen et al., 2017).

Also, a few studies reported enhanced processing of duration, both pre-attentively and attentively among musicians. For example, Marie et al. (2012) found larger pre-attentive and attentive responses to duration deviants among native speakers of Finnish, a language with phonemic vowel length contrast, and French musicians relative to non-musicians. In another study, Chobert et al. (2014) found that both the passive and the active processing of vowel duration and voice-onset-time (VOT) deviants were enhanced in musicians compared with non-musician children. These findings suggested that linguistic and musical expertise similarly influenced the processing of pitch contour and duration in music and language, possibly because they tapped on the same pool of neural resources (Besson et al., 2011). The duration effect was mostly examined on native speakers or non-tonal speakers. Therefore, it is worth examining whether similar duration effect can be observed among musicians and non-musicians with a tone language background.

In addition to stimulus duration, intrinsic *F*_0_ effects have been recently reported to contribute to CP (Chen et al., 2017). Intrinsic *F*_0_ effects refer to the consistent correlation between *F*_0_-values and vowel height (Whalen and Levitt, 1995). In speech production, high vowels are correlated with higher *F*_0_-values and low vowels with low *F*_0_-values. But such correlation is reversed in speech production, namely, high vowels are perceived to have a lower *F*_0_-value when they actually share the same *F*_0_ (Wang et al., 1976; Stoll, 1984). Yu et al. (2014) also proposed a hypothesis that if perceptual compensation occurs for high vowels, where they are perceived to have a lower *F*_0_-value, then high vowels may in turn be perceived as longer. Chen et al. (2017) found that Mandarin and English listeners required a longer duration to perceive a tone on a low vowel than a high vowel. Chen et al. (2020) also reported that vowel quality significantly contributed to tone identification and sharpness of category boundary in English and Mandarin musicians. Vowel quality also plays a role in tone perception especially for musicians and they were better at teasing apart the factor of vowel quality that may affect the *F*_0_ cue than non-musicians. The current study aims to examine the factor of vowel quality in tone perception by non-native tonal speakers with and without musical experience.

### The Current Study

We have three research goals for the current study: (1) to investigate the effects of musical experience on CP of pitch in a non-native language by native speakers of a tone language; (2) to examine how longer stimulus duration affects auditory categorization of pitch among non-native tonal musicians and non-musicians; (3) to investigate if musicians are more sensitive to factors that may potentially influence pitch processing such as vowel quality and pitch directions than non-musicians.

## Methodology

### Participants

A total of 28 native speakers of Cantonese participated in the experiment. All participants speak Cantonese as the first and dominant language and they also speak English and Mandarin. Of all the participants, 14 were musicians (seven males, seven females; mean age ± SD: 23.79 ± 2.40; age range: 19–27) and the other 14 were non-musicians (seven males, seven females; mean age ± SD: 23.86 ± 2.70; age range: 19–27). The musicians began receiving formal musical training of western music instruments at an average age of 6.96 (± 1.95) years and all had regular practice of the instruments at the time of the experiment (mean years of musical experience ± SD: 16.80 ± 3.31; range: 10–23). The non-musicians did not receive any after-school musical training. To confirm that the two groups of participants had similar background of Mandarin learning, we further collected information on the onset age of Mandarin learning, years of Mandarin learning as well as self-reported proficiency of reading, writing, listening, and speaking abilities in Mandarin on a five-point scale as listed in Table 1 (Point 1 indicates the lowest level of proficiency and point 5 indicates the highest level of proficiency). The participants reported no history of speaking, hearing, or language difficulty.

**Table 1:** Participants' background information.

**Factor**	**Musician**	**Non-musician**
Age	23.79 ± 2.40	23.86 ± 2.70
Onset age of musical training	6.96 ± 1.95	N/A
Years of musical training	16.80 ± 3.31	N/A
Onset age of Mandarin learning	5.43 ± 2.26	5.79 ± 1.82
Years of Mandarin learning	7.85 ± 2.56	8.14 ± 2.20
Self-reported reading ability in Mandarin	4.57 ± 0.49	4.71 ± 0.45
Self-reported writing ability in Mandarin	4.43 ± 0.73	4.36 ± 0.48
Self-reported listening ability in Mandarin	3.64 ± 1.11	3.71 ± 0.45
Self-reported speaking ability in Mandarin	3.64 ± 0.72	3.50 ± 0.50

### Stimuli

To examine the role of vowel quality, two sets of stimuli were created in the same way on low and high vowels [a] and [i]. A male native speaker of Mandarin with no reported speaking or hearing problem produced the Mandarin syllables [a] and [i] with the high-level Tone 1 using an Audio-Technica AT2020 microphone in a soundproof booth of the phonetics lab at the University of Florida. For each set of stimuli, the pitch contour of the original target syllable was manipulated with the pitch synchronous overlap add (PSOLA) method (Moulines and Laroche, 1995) in Praat (Boersma and Weenink, 2015). Our stimuli were all linear, and the slope and intercept parameters followed the estimates of a previous study based on a corpus of Mandarin speech (Prom-on et al., 2009). According to Prom-on et al. (2009), the slope and intercept for the rising Tone 2 in Mandarin are 93.4 and −2.2 st, respectively. Equation (1) below was used to transform st from Hertz (Hz):


(1) $$\begin{array}{r} Number\;of\;st=\frac{12}{\log_{10}2}*\log\left(F_{02}/F_{01}\right) \end{array}$$

where *F*_01_ and *F*_02_ represent the lower *F*_0_ and the higher *F*_0_, and the number of st measures the distance between *F*_01_ and *F*_02_ in Hz (Lehnert-LeHouillier, 2013). Following Xu et al. (2006), we set the *F*_02_-value at 130 Hz, and calculated the *F*_01_-value based on the chosen *F*_02_-value and the intercept value of −2.2st.

**Figure 1:**
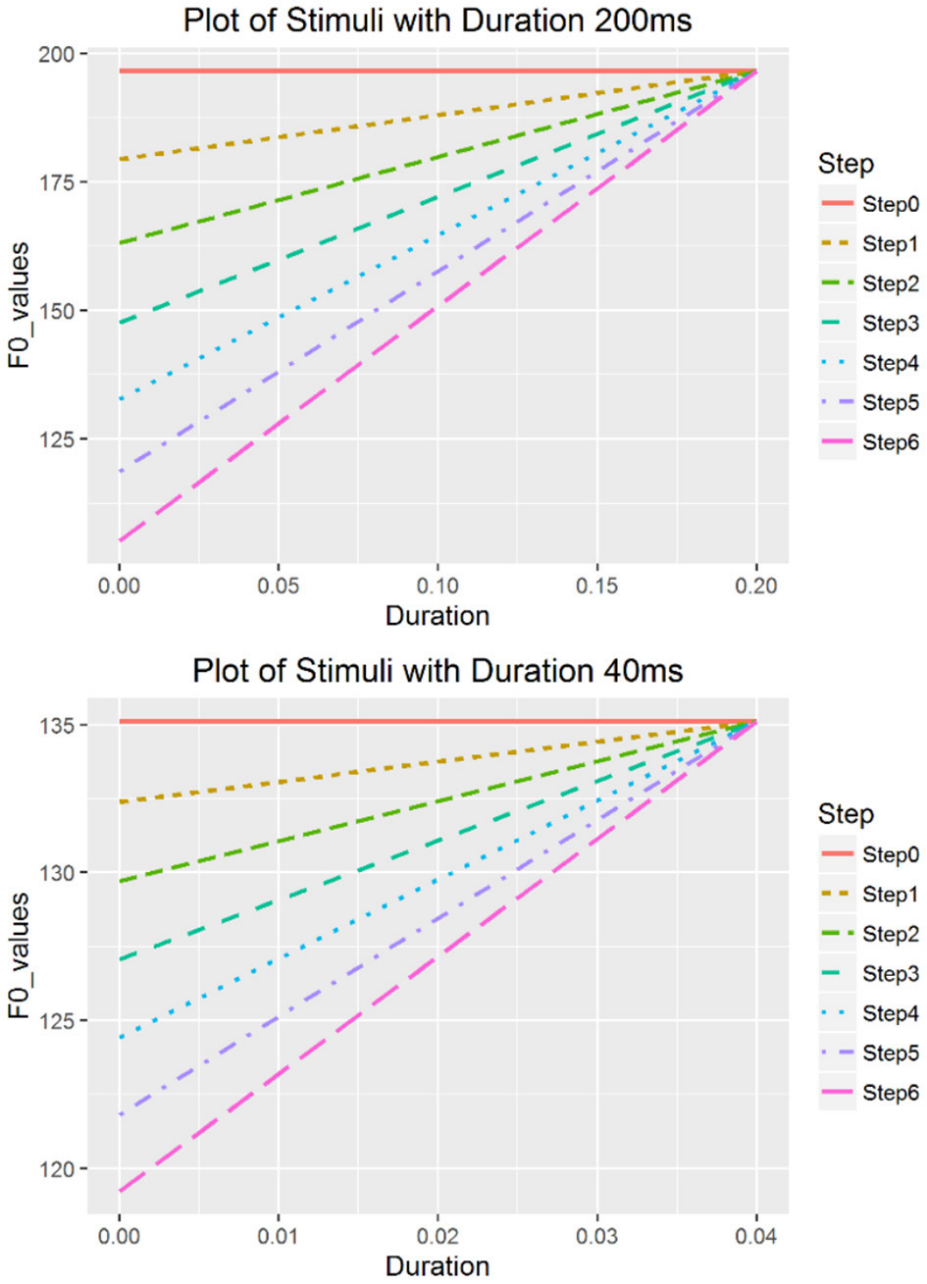
Rising continua with duration of 200 ms (top) and 40 ms (bottom).

**Figure 2:**
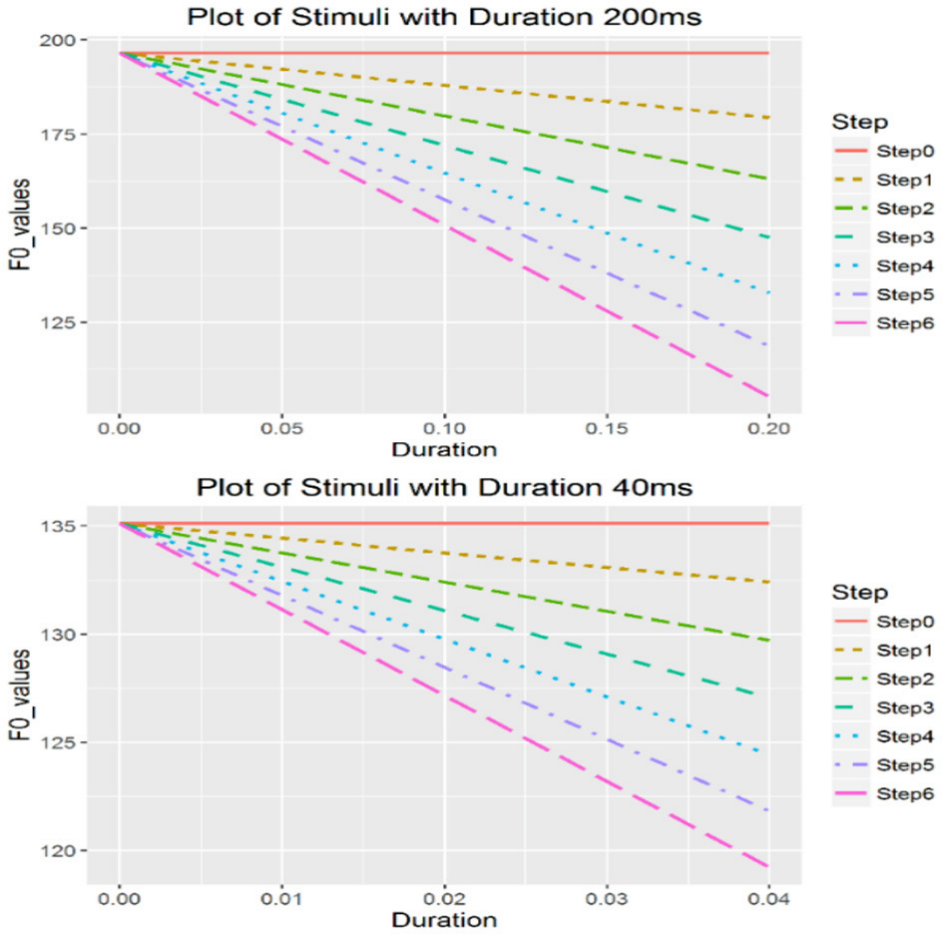
Falling continua with duration of 200 ms (top) and 40 ms (bottom).

For each vowel, nine durations were manipulated: 200, 180, 160, 140, 120, 100, 80, 60, and 40 ms. In total, there were 18 continua (9 duration * 2 vowels). The stepwise onset values with different duration values for the rising tone are as shown in Table 2 and those for the falling tone are listed in Table 3. Figures 1, 2 are examples of two rising continua with the durations of 200 and 40 ms.

**Table 2:** Onset values for each step varied by duration for linear rising pitch directions.

**Duration**	**0.2**	**0.18**	**0.16**	**0.14**	**0.12**	**0.1**	**0.08**	**0.06**	**0.04**
Step 0	196.56	187.56	178.97	170.78	162.96	155.50	148.38	141.59	135.11
Step 1	179.43	172.62	166.09	159.84	153.86	148.14	142.66	137.42	132.40
Step 2	163.12	158.31	153.69	149.26	145.02	140.94	137.04	133.30	129.72
Step 3	147.58	144.61	141.76	139.03	136.41	133.91	131.52	129.24	127.06
Step 4	132.77	131.49	130.27	129.13	128.05	127.04	126.11	125.24	124.43
Step 5	118.67	118.92	119.21	119.55	119.92	120.33	120.79	121.28	121.82
Step 6	105.23	106.89	108.57	110.28	112.01	113.78	115.57	117.39	119.24

**Table 3:** Offset values for each step varied by duration for linear falling pitch directions.

**Duration**	**0.2**	**0.18**	**0.16**	**0.14**	**0.12**	**0.1**	**0.08**	**0.06**	**0.04**
Step 0	196.56	187.56	178.97	170.78	162.96	155.50	148.38	141.59	135.11
Step 1	179.43	172.62	166.09	159.84	153.86	148.14	142.66	137.42	132.40
Step 2	163.12	158.31	153.69	149.26	145.02	140.94	137.04	133.30	129.72
Step 3	147.58	144.61	141.76	139.03	136.41	133.91	131.52	129.24	127.06
Step 4	132.77	131.49	130.27	129.13	128.05	127.04	126.11	125.24	124.43
Step 5	118.67	118.92	119.21	119.55	119.92	120.33	120.79	121.28	121.82
Step 6	105.23	106.89	108.57	110.28	112.01	113.78	115.57	117.39	119.24

Using a rising continuum as an example, we describe how steps in a continuum are calculated as follows. First, the offset values for each duration were calculated with Equation (2):


(2) $$\begin{array}{r} X\left(t\right)=93.4*t-2.2 \end{array}$$

where *t* is the duration in s, and X(*t*) stands for the semitone (st)-values at the offset of the tone. For example, for the 200 ms continuum, *t* = 200 ms (0.2 s) and the onset can be calculated by setting *t* = 0 s, which is −2.2 st (or 123.02 Hz); and the offset can be calculated by setting *t* = 0.2 s, which is 16.48 st (or 196.56 Hz). Therefore, the onset-to-offset distance [i.e., Δ*X*(*t*)] is 16.48 – (−2.2) = 18.68 st in this case. Stimuli with various onsets were created based on the calculated intercept value of −2.2 st, which was also the cutoff point. The extreme points of onset values were then determined so that the distance between the lowest onset value (−8.43 st or 105.23 Hz) and −2.2st was one third of the distance between the highest onset value (16.48 st or 196.56 Hz) and −2.2st. The highest onset was defined as the same value as the obtained offset value (196.56 Hz) and is used to generate a level tone with equal onset and offset values. After obtaining the highest and lowest onsets, steps were created between them based on the ERB scale instead of Hertz because the former reflects natural perception (Xu et al., 2006). Seven stimuli with equal perceptual distance were created for each duration value in a continuum, and the onset values were then transformed back into Hertz.

Our resynthesizing procedure is similar to Peng et al. (2010): (1) we adjusted the duration of the stimuli to the duration values in Tables 1, 2; (2) we peak normalized the stimuli to the same intensity level; (3) We adjusted the pitch points according to the values in Tables 1, 2. The set of stimuli used by Chen et al. (2020) was used in this current study.

For the identification task, stimuli with a rising pitch continuum were grouped in one block and those with a falling pitch continuum were grouped in another block. There were 630 stimuli (5 repetitions * 7 steps * 9 duration * 2 syllable) in each block.

Since a one-step difference is too difficult to perceive (Francis et al., 2003), so this study used two-step difference pairs for the same-difference discrimination task. Again, the stimulus presentation was blocked by rising and falling pitch directions. Within each block, the different pairs were presented in either the forward order (0–2, 1–3, 2–4, 3–5, 4–6) or the backward order (2–0, 3–1, 4–2, 5–3, 6–4). All the same and different pairs were repeated twice in the task. Overall, there were 612 trials in each block [9 durations * (7 same pairs + 10 different pairs) * 2 syllables * 2 repetitions].

### Procedure

All the participants signed informed consent forms in compliance with a protocol approved by the Human Subjects Ethics Sub-committee at the Hong Kong Polytechnic University and participated in the experiment with a GMH C 8.100 D headset at the Speech and Language Sciences Lab of the Hong Kong Polytechnic University. An identification task and a same-difference discrimination task were implemented in E-Prime 2.0 (Schneider et al., 2012). There was one practice block and two test blocks for each task where the practice block always preceded the test block. Within each block, the order of stimulus presentation was randomized. The order of block presentation for each participant was also counterbalanced and the block order was kept identical for the musician group and the non-musician group.

#### Training and Practice

Prior to the actual tasks, the participants were first familiarized with the tasks and the participants were required to identify the pitch directions they heard by pressing the number key 1 for a level tone and the number key 2 for a rising or falling tone in a practice session of the identification task. In the practice session for the same-difference discrimination task, the participants listened to a pair of stimuli at a time and were asked to decide whether the stimuli had the same or different pitch direction by pressing the number key 1 for “the same” and the number key 2 for “different.” Only stimuli with the longest duration value (0.2 s) at the two endpoints of each continuum were used for the practice sessions. A minimum threshold [75% (12 out of 16 trials); 71% (17 out of 24 trials) for the identification and the discrimination tasks, respectively] of correct responses was set for each practice session to make sure that the participants were able to finish the tasks and can identify or discriminate the pitch directions with either a level or steep rising/falling tone with the longest duration. Only those who passed the threshold proceeded to the experimental tasks. The reasons for including the practice session is that we need to make sure that the participants are familiar with the procedure required by the tasks and are able to press the right keys when listening to the pitch directions.

#### The Identification Task

In the identification task, the participants followed the same procedure as in the practice session. Once they heard a stimulus, they needed to decide whether it was a level tone or a rising/falling tone by pressing “1” for the former and “2” for the latter at a self-paced rate. The next stimulus was presented automatically after a response was given.

#### The Same-Different Discrimination Task

In the discrimination task, the participants listened to a pair of stimuli in each trial and were asked to decide whether the two stimuli were the same or different in terms of pitch direction at a self-paced rate. There were 34 trials of stimuli for each duration value [(7 same pairs + 10 different pairs) * 2 repetitions]. The 34 trials were divided into five two-step comparison units: 0–2, 1–3, 2–4, 3–5, and 4–6, where each unit had four types of comparisons: AA, AB, BA, and BB. The same trials were included. For example, the 3–3 pair was included in both 1–3 and 3–5 units. Therefore, there were eight trials (4 types of comparison * 2 repetitions) for each unit. In addition, the ISI of 500 ms was used as previous research suggests that 500 ms is the time needed to maximize the differences in between- vs. within-category discrimination (Pisoni, 1973; Xu et al., 2006; Peng et al., 2010).

The stimuli were presented automatically after a response was given. For further analyses, *d*-prime (*d*′) scores were computed from raw discrimination responses with the Equation (3):


(3) $$\begin{array}{r} d^{\prime }=z\left(H\right)-z\left(F\right) \end{array}$$

where *d'* is the *d*-prime score, *H* is the hit rate (i.e., “different” response given for “different” trials), *F* is the false alarm rate (i.e., “different” response given for “same” trials) and *z* is *z*-transform (Creelman and Macmillan, 2004).

### Data Analysis

There are three features of CP, namely a sharp category boundary, a discrimination peak, and prediction of discrimination from identification. The data obtained were, therefore, analyzed to examine the effects of musical background (musicians vs. non-musicians), pitch direction (rising vs. falling), vowel quality (low vs. high), and duration (nine different duration values) on these characteristics of CP.

#### The Identification Task

The identification data was analyzed to see if category boundary sharpness and category boundary location were affected by pitch direction type (falling vs. rising), vowel quality (low [a] vs. high [i]), stimulus duration (nine values: 40–200 in 20 ms increment) and musicianship (musicians vs. non-musicians). To achieve these goals, a generalized linear mixed model with subjects as a random effect was fitted to the data using the lme4 package (Bates et al., 2015) in R (R Core Team, 2018).

To perform the analyses, the data were divided into eight subgroups based on musical background, pitch direction, and vowel quality: FCMA, FCMI, RCMA, RCMI, FCNA, FCNI, RCNA, and RNI, where F and R represent falling and rising pitch directions, CM and CN stand for Cantonese musicians and non-musicians, and A and I for [a] and [i] syllables. For example, RCNA is the subset of data which includes rising pitch direction (R) on the syllable [a] (A) identified by Cantonese non-musicians (CN). Within each subgroup, a generalized linear mixed model was fitted with identification scores (0 or 1) as the response variable (the accuracy rate can be calculated from 0 and 1 s) and step number (*x* = 0–6) as a factor. The model is similar to a logistic regression model when only the fixed effects are considered in Equation (4).


(4) $$\begin{array}{r} \log_{e}\left(\frac{p_{1}}{1-p_{1}}\right)=b_{0}+b_{1}x \end{array}$$

In this equation, the coefficient *b*_1_ stands for the sharpness of category boundary. To perform a *post-hoc* analysis on the effects of sharpness of boundary by duration, we carried out pairwise comparisons between different duration values for each of the eight subgroups. Specifically, we fit a model treating the coefficient *b*_1_ from each pair of duration values as the same and conducted a likelihood ratio test to compare it to another model that treats them as different. Significant differences between the two models would indicate significant differences of the coefficient *b*_1_ between two duration values. We also tested whether musical background, pitch direction and vowel quality would influence the values of *b*_1_. In addition, we modeled the relationship between the sharpness of category boundary and duration for musicians and non-musicians.

For category boundary location, after obtaining the estimates for *b*_0_ and *b*_1_, *P*_1_ = 0.5 was used to estimate the step number at category boundary within musician and non-musician groups, as shown in Equation (5):


(5) $$\begin{array}{r} x_{cb}=-b_{0}/b_{1} \end{array}$$

Once the individual category boundary was obtained for subjects in each subgroup, a linear mixed effects model was fitted, with pitch direction, musicianship, duration, and vowel quality as fixed effects and subjects as random effects, followed by *post-hoc* analyses. Linear regression models were fitted separately for musicians and non-musicians to test the relationship between duration and category boundary.

Finally, formulas for minimum stimulus duration required to perceive a rising or a falling *F*_0_ from a level *F*_0_ were derived using Equation (6):


(6) $$\begin{array}{r} t=b_{0}+b_{1}d \end{array}$$

where *t* is the duration needed to perceive *d* st differences from level tones. For each duration value, the estimated step (identification rate equaled 0.5) was recorded and set as a cut-off point. Step numbers smaller than this point indicated that the stimulus was more likely to be identified as a level tone. The step number was transformed back to st-values with respect to the baseline (level tone as step 0) for each duration value. Linear mixed effects models were then fitted to obtain a relationship between cut-off st-values and duration, and it was assumed that the cut-off st-values were the smallest values for a rising or falling pitch direction to be perceived as different from a level tone. Minimum stimulus duration formulas for each of the eight subgroups were derived separately, and more general formulas for musicians and non-musicians to perceive each pitch direction were also obtained.

#### The Discrimination Task

*d*-prime's scores based on correct (hits) and incorrect (false alarms) responses were obtained in the discrimination task, and a generalized linear mixed model were fitted with duration, musical background, and vowel quality and all the two-way and three-way interactions as fixed factors and subject as a random factor.

To explore the relationship between between-and within-category discrimination, *d*-prime scores of between- and within-category discrimination for each subgroup according to its category boundary was calculated (Wu et al., 2015). The *d*-prime scores were calculated for all nine duration values. When the category boundary was <1 or >5, the *d*-prime score was not calculated, because the steps were constrained between 0 and 6 steps. Linear mixed-effects models were then fitted to examine the contribution of musical background, pitch direction, vowel quality, and duration to the *d*-prime scores, followed by *post-hoc* analyses. Pairwise comparison of duration was performed, and linear regression models were fitted to examine the relationship between discrimination scores and duration.

The peakedness of discrimination function was estimated from the difference between *P*_*bc*_ and *P*_*wc*_. *P*_*bc*_ (between-category discrimination) is defined as *P* of the comparison unit corresponding to the category boundary, and *P*_*wc*_ (within-category discrimination) is defined as the average of two comparison units at the extremes of the continuum (*P*_02_ and *P*_46_) (Pisoni, 1973). Linear models were fitted to examine whether there were any significant contributing factors. Pairwise comparison of duration with respect to peakedness was conducted, and regression models were also fitted to investigate the relationship between peakedness and duration.

#### Predicting Discrimination From Identification

According to Pollack and Pisoni (1971), the predicted discrimination score P* can be calculated by Equation (7):


(7) $$\begin{array}{r} P^{*}=\left[1+{\left(P_{A}-P_{B}\right)}^{2}\right]/2 \end{array}$$

where *P*_*A*_ and *P*_*B*_ are the identification scores in a comparison unit. This equation assumes that the discrimination can be solely determined by the identification of the two stimuli *A* and *B*. The correlation between the predicted and the observed discrimination scores for each comparison subgroup was calculated based on different trials of stimuli *A* and *B* by optimizing linear regression models after Fisher's *z* transformation. Next, the effects of musical background, pitch direction, vowel quality, and duration on the correlation was tested. The mean difference between the predicted and observed discrimination scores P – P^*^ were also calculated. The optimized linear model was selected based on the stepwise optimization algorithm using the function “step” (Hastie and Pregibon, 1992) to model the relationship between the distance and the tested variables.

## Results

### Identification

A generalized linear mixed effects model was fitted to the pitch direction (i.e., level vs. rising/falling) identification data obtained from Cantonese listeners with and without musical experience. The dependent variable was response rate, and the independent variables included vowel quality, pitch direction, duration, musical experience, and their interactions. The results revealed significant main effects of vowel quality [$\chi_{\left(1\right)}^{2}$ = 26.503, *p* < 0.001] and pitch direction [${\text{χ}}_{\left(1\right)}^{2}$ = 104.9, *p* < 0.001] as well as a marginally significant main effect of duration [${\text{χ}}_{\left(1\right)}^{2}$ = 3.285, *p* = 0.07]. The main effect of musical experience, however, did not reach significance [${\text{χ}}_{\left(1\right)}^{2}$ = 1.017, *p* = 0.313]. These results indicated that a syllable with the high vowel [i] was more likely to be identified as a level tone than a syllable with the low vowel [a], and a rising syllable was more likely to be identified as a level than a falling syllable. The chance of a syllable being identified as a level tone also decreased with the increase of syllable duration. The two-way interactions were significant for vowel quality and musical experience [${\text{χ}}_{\left(1\right)}^{2}$ = 9.356, *p* = 0.002], musical experience and pitch direction [${\text{χ}}_{\left(1\right)}^{2}$ = 21.465, *p* < 0.001], and musical experience and duration [${\text{χ}}_{\left(8\right)}^{2}$ = 169.99, *p* < 0.001]. *Post-hoc* tests suggested that (1) vowel quality did not influence the identification of non-musicians, but musicians were more likely to identify a high vowel /i/ as a level tone; (2) both groups of participants were more likely to identify a falling syllable as a level tone; (3) with the increase of duration, musicians tended to identify a syllable as a rising/falling tone, while non-musicians tended to identify a syllable as a level tone.

#### Sharpness of Category Boundary

**Figure 3:**
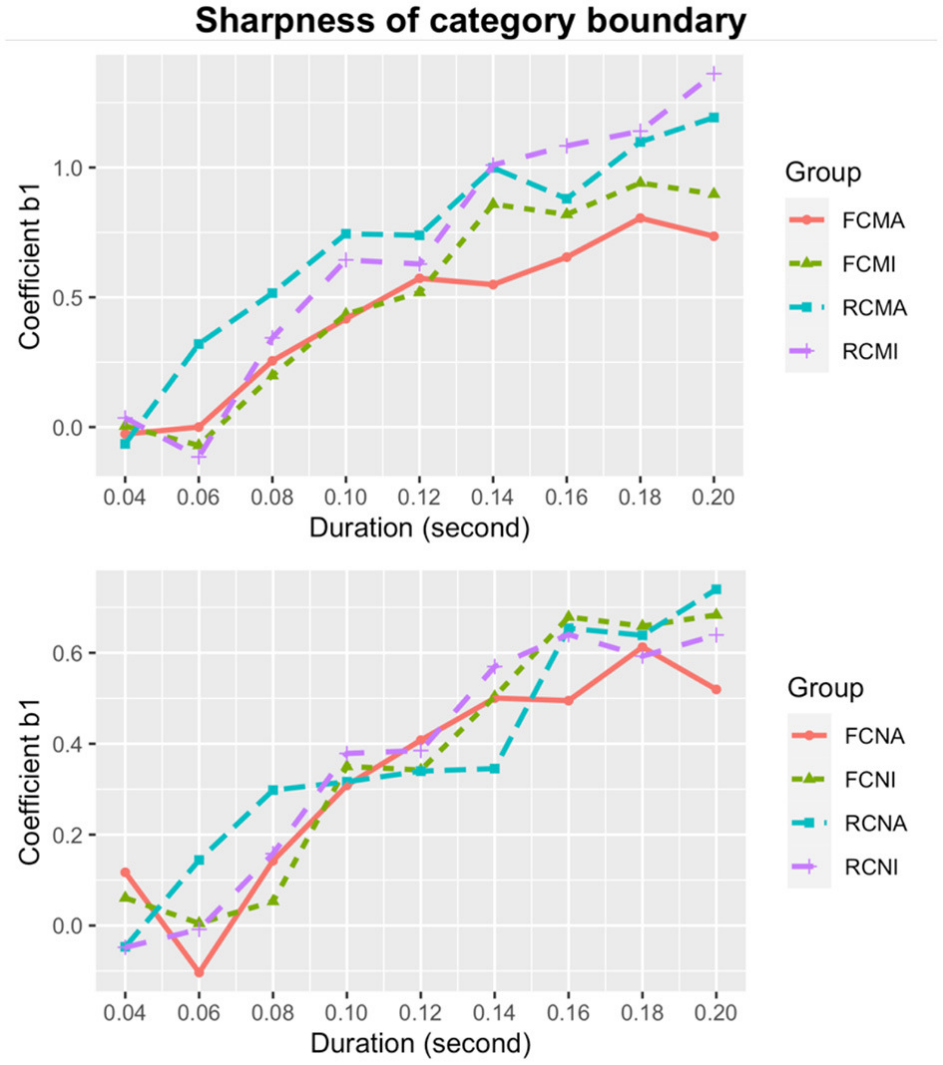
Sharpness of category boundary for Cantonese musicians (upper) and non-musicians (lower).

The estimates of the coefficient *b*_1_ (sharpness of category boundary) of all nine stimulus durations for each subgroup are plotted in Figure 3. From Figure 3, it is evident that category boundary becomes sharper as the stimulus duration increases for all subgroups. In addition, overall, the musicians showed a sharper category boundary than the non-musicians.

We extracted all the values of category boundary sharpness *b*_1_ and treat *it* as the dependent variable. The independent variables included vowel quality, pitch direction, duration, musical experience, and their interactions. Likelihood ratio tests revealed significant effects of musical experience [${\text{χ}}_{\left(1\right)}^{2}$ = 97.687, *p* < 0.001]; pitch direction [${\text{χ}}_{\left(1\right)}^{2}$ = 26.733, *p* < 0.001]; and vowel quality [${\text{χ}}_{\left(1\right)}^{2}$ = 26.503, *p* < 0.001]. These results suggested that, on average, category boundary was significantly sharper for musicians than non-musicians [*b*_1_ = 0.587 vs. 0.363]; for the rising pitch than the falling pitch direction [*b*_1_ = 0.536 vs. 0.414], and for the high vowel /i/ than the low vowel /a/ [*b*_1_ = 0.483 vs. 0.483]. There were also significant interactions between musical experience and pitch direction [${\text{χ}}_{\left(3\right)}^{2}$ = 150.47, *p* < 0.001], between musical experience and vowel quality [${\text{χ}}_{\left(3\right)}^{2}$ = 110.48, *p* < 0.001], and between musical experience and duration [${\text{χ}}_{\left(10\right)}^{2}$ = 3686.4, *p* < 0.001]. For both groups of listeners, follow up tests revealed higher *b*_1_ (sharper category boundary) values for the rising than the falling pitch directions and for the high vowel [i] than the low vowel [a]. However, none of the differences reached statistical significance.

To examine the significant interaction between musicianship and stimulus duration on category boundary sharpness, regression models were separately fitted for musicians and non-musicians. For the musicians, a regression model with an extra quadratic term did not significantly improve the model compared to a linear regression model, as suggested by a likelihood ratio test [${\text{χ}}_{\left(2\right)}^{2}$ = 0.187, *p* = 0.911]. The linear regression model in Equation (8) was thus selected and it captured the relationship between the duration and sharpness well [*F*_(1,34)_ = 153.1, *p* < 0.001; adjusted *R*^2^ = 0.813]. For the non-musicians, a regression model with an extra quadratic term did not significantly differ from a linear regression model [${\text{χ}}_{\left(2\right)}^{2}$ = 2.214, *p* = 0.331], so the linear model in Equation (9) was adopted [*F*_(1,34)_ = 251.7, *p* < 0.001; adjusted *R*^2^ = 0.878].


(8) $$\begin{array}{r} b1=7.067*d-0.261 \end{array}$$

(9) $$\begin{array}{r} b1=4.498*d-0.177 \end{array}$$

The formulae show that the sharpness of category boundary for both Cantonese musicians and non-musicians increases with the stimulus duration, but the musicians exhibit a steeper slope and thus have a faster increment of sharpness of category boundary with increased duration.

#### Category Boundary Location

**Figure 4:**
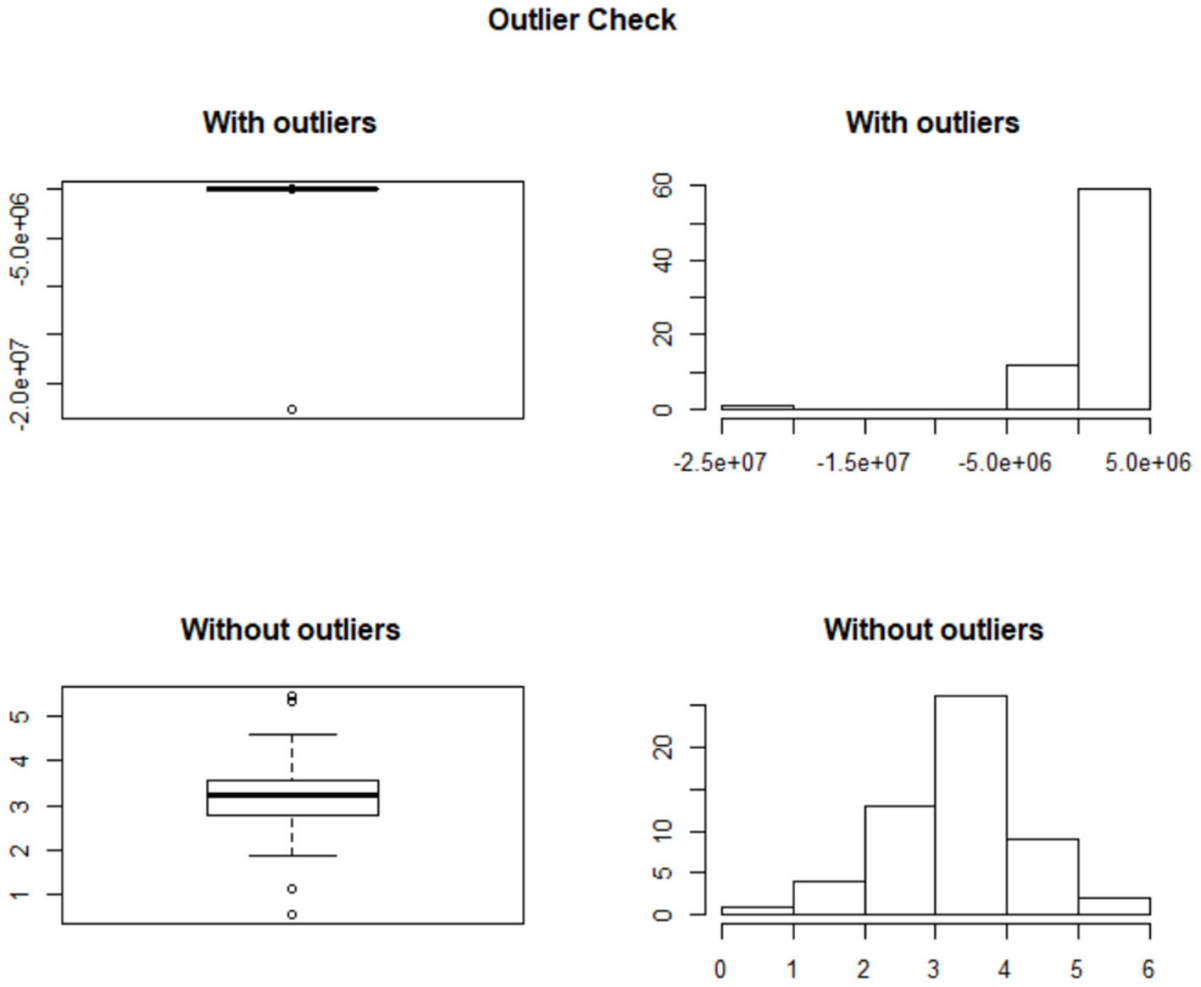
Plots with and without outliers. The upper figures present the original data, which are severely left-skewed. The right figures represent the data after removing the outliers, which are normally distributed.

**Figure 5:**
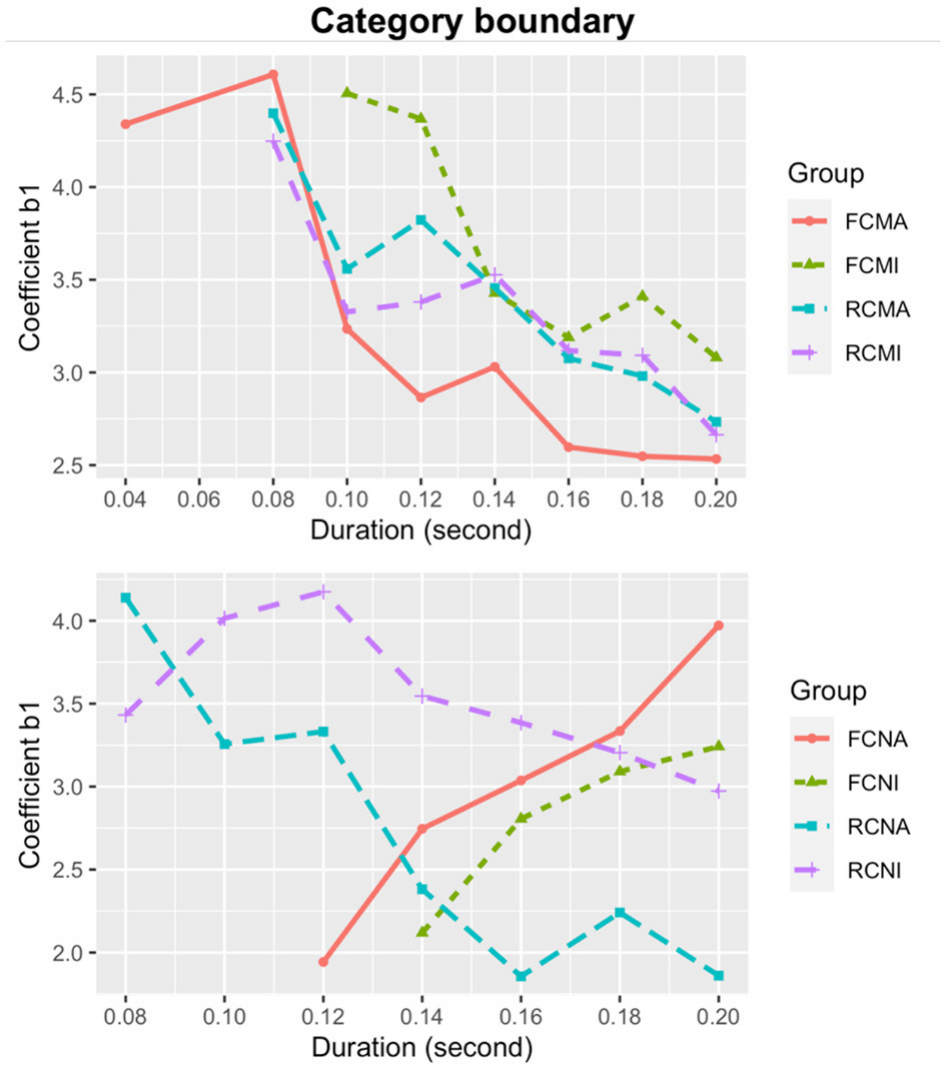
Category boundary for Cantonese musicians and non-musicians. The upper figure shows the category boundary of the musician group and the lower one stands for that of the non-musician group.

Category boundary for each subgroup and each duration value was calculated based on the estimated coefficients *b*_0_ and *b*_1_ from the generalized linear models. After removing all the outliers^1^ as illustrated in Figure 4, we plotted the category boundary against stimulus duration for each subgroup in Figure 5.

In general, we see that category boundary shifted to smaller values as the stimulus duration increased. A linear regression model was fitted, where the dependent variable was category boundary, and the independent variables included musicianship, vowel quality, duration, and pitch direction. A significant effect of stimulus duration (*p* < 0.001) was found, but a marginal effect of musical experience was identified (*p* = 0.068), wherein boundary locations were smaller for non-musicians than musicians. No effects of vowel quality (*p* = 0.143) or pitch direction (*p* = 0.846) were found.

To capture the relationship between category boundary location and stimulus duration for Cantonese musicians and non-musicians, regression models were fitted to the data. For the musicians, no significant difference was observed between a regression model with only the slope and intercept terms and a regression model with an extra quadratic term, as suggested by a likelihood ratio test [${\text{χ}}_{\left(1\right)}^{2}$ = 1.793, *p* = 0.181], so the simple model [*F*_(1,24)_ = 33.77, *p* < 0.001; adjusted *R*^2^ = 0.567] was adopted [Equation (10)]. Similarly, for the Cantonese non-musicians, no significant difference was found between a simple model and a complex model [${\text{χ}}_{\left(1\right)}^{2}$ = 2.328, *p* = 0.127], so the simple model [*F*_(1,19)_ = 0.495, *P* = 0.490; adjusted *R*^2^ = −0.026] was adopted [Equation (11)].


(10) $$\begin{array}{r} cb=-12.008*d+5.068 \end{array}$$

(11) $$\begin{array}{r} cb=-3.409*d+3.509 \end{array}$$

We can infer from the equations that the category boundary shifted to smaller values as the stimulus duration increases, and the decrease rate is faster for the musician group.

### Same-Different Discrimination Task

The raw data of the same-different discrimination task were transformed into a response of either 0 (same) or 1 (different) to fit generalized linear mixed effects models to test the main effects and interaction terms. The dependent variable was the response variable, and the independent variables included musicianship, vowel quality, duration, and pitch direction and their interactions. The results revealed significant main effects of vowel quality [${\text{χ}}_{\left(1\right)}^{2}$ = 6.451, *p* = 0.011], pitch direction [${\text{χ}}_{\left(1\right)}^{2}$ = 583.31, *p* < 0.001], and duration [${\text{χ}}_{\left(1\right)}^{2}$ = 2507.2, *p* < 0.001]. The main effect of musical experience did not reach significance [${\text{χ}}_{\left(1\right)}^{2}$ = 2.377, *p* = 0.123]. The two-way interactions were significant for vowel quality and musical experience [${\text{χ}}_{\left(2\right)}^{2}$ = 27.002, *p* < 0.001], vowel quality and pitch direction [${\text{χ}}_{\left(2\right)}^{2}$ = 10.417, *p* = 0.005], vowel quality and duration [${\text{χ}}_{\left(1\right)}^{2}$ = 4.867, *p* = 0.027], musical experience and pitch direction [${\text{χ}}_{\left(1\right)}^{2}$ = 21.098, *p* < 0.001], and music experience and duration [${\text{χ}}_{\left(1\right)}^{2}$ = 4.079, *p* = 0.043]. *Post-hoc* tests showed the following: (1) the musicians tended to regard the low vowel [a] stimuli as being the same compared to the high vowel [i], while the non-musicians had the reversed pattern; (2) the musicians were more likely to regard the stimuli as different than the non-musicians, regardless of duration or pitch direction; (3) the low vowel [a] stimuli were more likely to be regarded as the same compared to the high vowel [i] stimuli, regardless of the pitch direction or duration; (4) the rising pitch direction was more likely to be treated as the same compared to the falling pitch direction, regardless of duration values.

#### Between-Category Discrimination

For each subject, the *d*-prime scores of the comparison unit corresponding to the category boundary were calculated. Linear mixed-effects models were then fitted with *d*-prime scores as the response variable, musical experience, pitch direction, vowel quality, and duration as factors, and subjects as a random effect. The main effects of musical experience [${\text{χ}}_{\left(1\right)}^{2}$ = 7.486, *p* = 0.006], pitch direction [${\text{χ}}_{\left(1\right)}^{2}$ = 37.065, *p* < 0.001], and duration [${\text{χ}}_{\left(1\right)}^{2}$ = 33.938, *p* < 0.001] all reached significance. There were also significant two-way interactions between musical experience and pitch direction [${\text{χ}}_{\left(1\right)}^{2}$ = 29.447, *p* < 0.001], musical experience and vowel quality [${\text{χ}}_{\left(1\right)}^{2}$ = 6.495, *p* = 0.039], pitch direction and vowel quality [${\text{χ}}_{\left(1\right)}^{2}$ = 6.800, *p* = 0.033], and duration and vowel quality [${\text{χ}}_{\left(1\right)}^{2}$ = 7.962, *p* = 0.004]. *Post-hoc* tests suggested that musicians had higher *d*-prime scores than non-musicians regardless of pitch direction, duration, or vowel quality. Also, the falling pitch direction had higher *d*-prime scores than the rising pitch direction for musicians, while the reversed pattern was observed for non-musicians. In general, *d*-prime scores improved with the increase of duration for both musicians and non-musicians.

Next, we fitted regression models for Cantonese musicians and non-musicians to capture the relationship between between-category *d*-prime scores (dependent variable) and duration. The model for the musicians (Equation 12) was non-significant [*F*_(1,25)_ = 4.412, *p* = 0.459; adjusted *R*^2^ = 0.116], and the one for non-musicians (Equation 13) was significant [*F*_(1,18)_ = 8.014, *p* = 0.011; adjusted *R*^2^ = 0.270].


(12) $$\begin{array}{r} bcd=22.710*d-3.073 \end{array}$$

(13) $$\begin{array}{r} bcd=17.616*d-1.975 \end{array}$$

#### Within-Category Discrimination

Within-category discrimination *d*-prime scores were calculated for each subject. A linear mixed effects model was fitted where the dependent variable was *d*-prime scores, and the independent variables included musicianship, vowel quality, duration, and pitch direction. The results showed significant main effects of pitch direction [${\text{χ}}_{\left(1\right)}^{2}$ = 140.99, *p* < 0.001; mean d′ scores = 1.441 and 0.777 for falling and rising pitch directions, respectively], and a marginally significant main effect of stimulus duration [${\text{χ}}_{\left(1\right)}^{2}$ = 3.674, p = 0.055; mean d′ scores ranged from 0.988 to 1.240]. There was no significant effect of musical experience on the *d*-prime scores.

We then fitted regression models for Cantonese musicians and non-musicians to capture the relationship between within-category *d*-prime scores (dependent variable) and duration (independent variable). The regression model for the musicians as in Equation (14) was significant [*F*_(1,25)_ = 6.747, *p* = 0.015; adjusted *R*^2^ = 0.181]. The model for non-musicians as in Equation (15) did not reach significance [*F*(_1,18_) = 2.67, *p* = 0.120; adjusted *R*^2^ = 0.081]. In general, the musicians had higher scores than the non-musicians, although not all pairs showed significant differences.


(14) $$\begin{array}{r} wcd=0.812+5.886*d \end{array}$$

(15) $$\begin{array}{r} wcd=0.305+5.775*d \end{array}$$

#### Peakedness

Peakedness of discrimination function was estimated by calculating the difference between *P*_bc_ (between-category discrimination) and *P*_wc_ (within-category discrimination). The dependent variable is the peakedness value, and the independent variables included musical experience, pitch direction and stimulus duration. The main effects of musical experience [${\text{χ}}_{\left(1\right)}^{2}$ = 5.828, *p* = 0.016] and pitch direction [${\text{χ}}_{\left(1\right)}^{2}$ = 10.434, *p* = 0.001] and stimulus duration [${\text{χ}}_{\left(1\right)}^{2}$ = 12.412, *p* < 0.001] reached significance. The two-way interactions between musical experience and pitch direction [${\text{χ}}_{\left(1\right)}^{2}$ = 15.469, *p* < 0.001] and between pitch direction and duration [${\text{χ}}_{\left(1\right)}^{2}$ = 3.970, *p* = 0.046] were also significant. In general, the musicians had larger peakedness than the non-musicians regardless of vowel quality, or duration. The rising pitch direction was always greater in peakedness than the falling pitch direction. There was also a general trend of greater peakedness with the increase of duration for both groups of participants. Moreover, although musicians had larger peakedness than non-musicians for the falling pitch direction, the two groups had comparable peakedness for the rising pitch direction.

Since musical experience was shown to influence the peakedness, we fitted regression models for musicians and non-musicians separately. However, the models suggested that duration did not significantly contribute to the change in peakedness for either musicians [*F*_(1,18)_ = 3.341, *p* = 0.084; adjusted *R*^2^ = 0.110] or for non-musicians [*F*_(1,6)_ = 2.43, *p* = 0.170; adjusted *R*^2^ = 0.170]. The models are listed in Equations (16) (musicians) and (17) (non-musicians).


(16) $$\begin{array}{r} pk=0.339-6.170*d \end{array}$$

(17) $$\begin{array}{r} pk=-0.704+6.825*d \end{array}$$

#### Predicted and Obtained Discrimination

**Figure 6:**
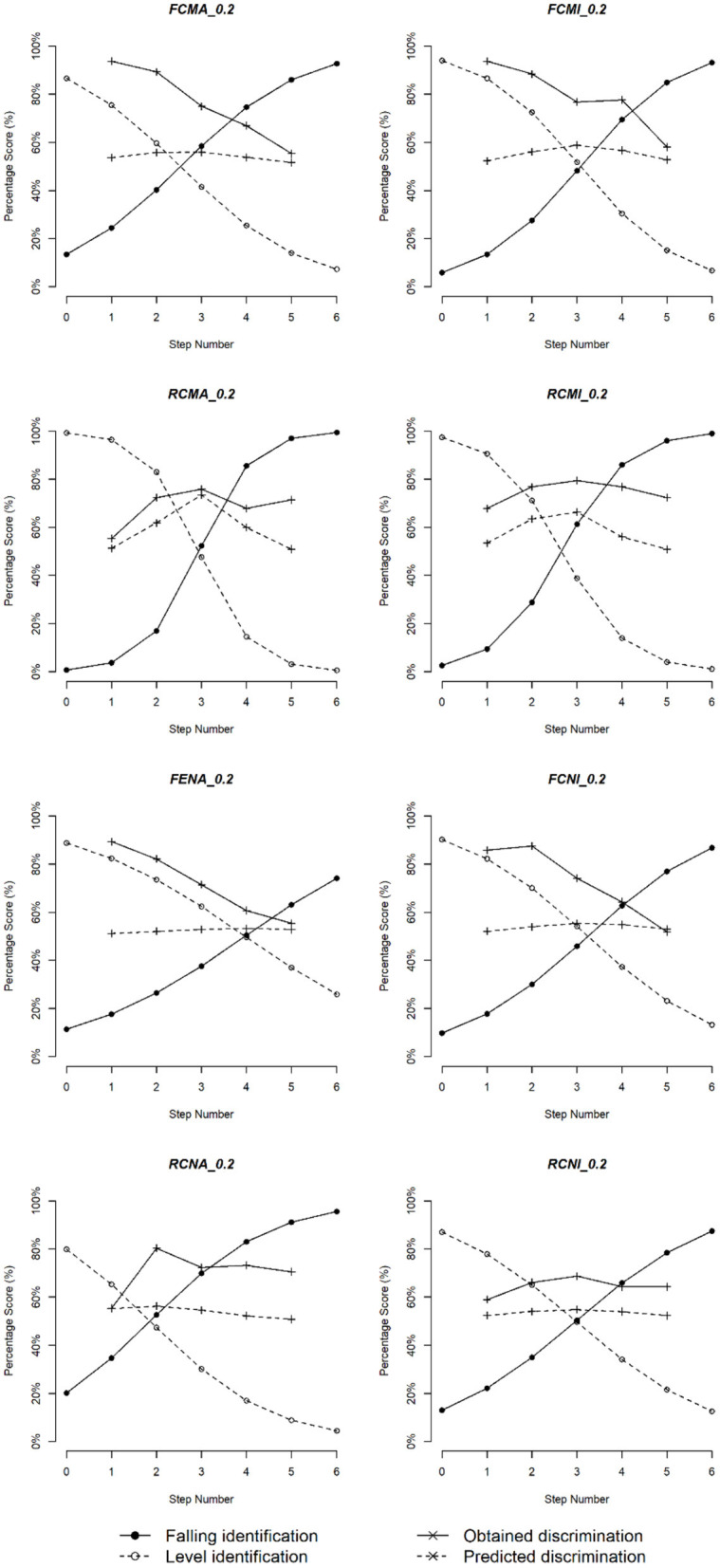
Logistic identification functions and discrimination curves for eight subgroups of Cantonese speakers with duration 200 ms.

**Figure 7:**
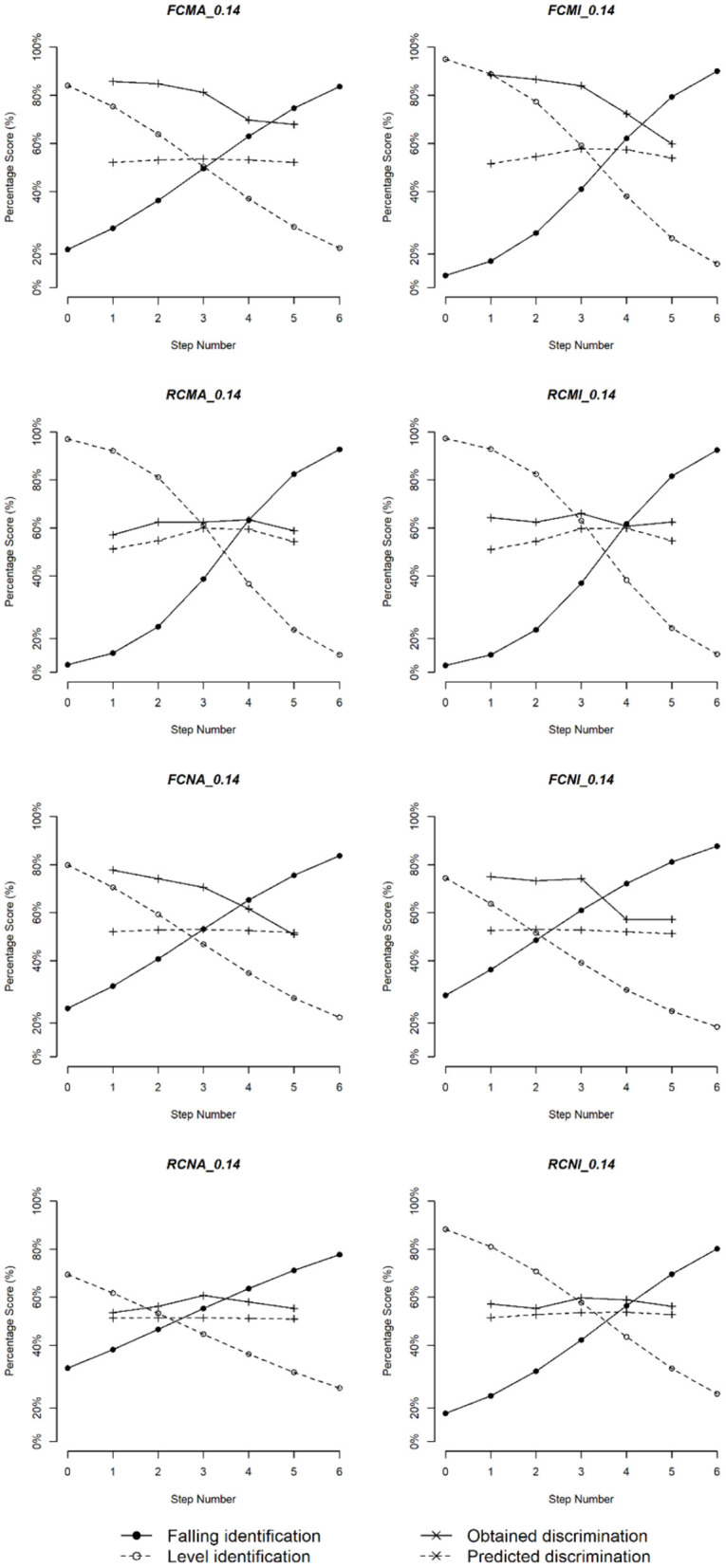
Logistic identification functions and discrimination curves for eight subgroups of Cantonese speakers with duration 140 ms.

**Figure 8:**
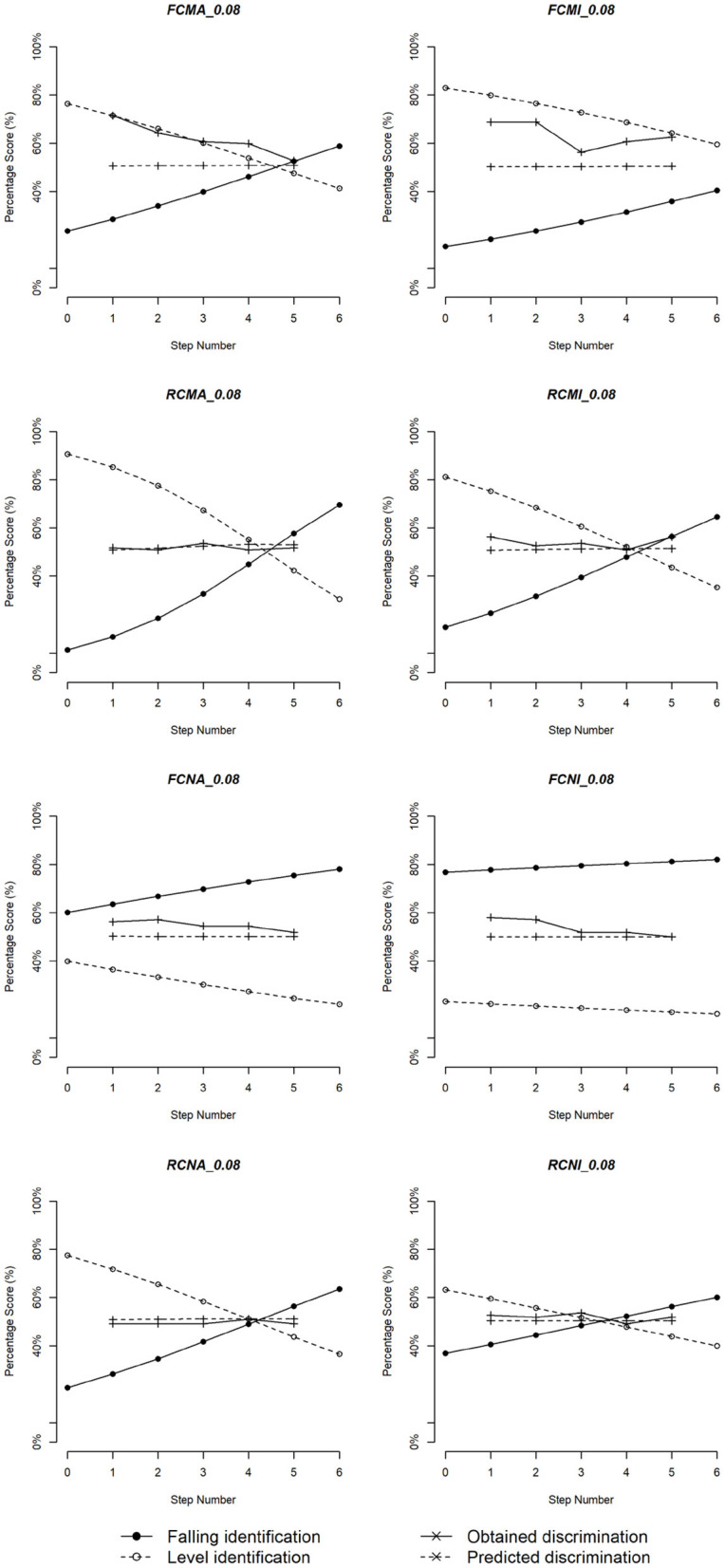
Logistic identification functions and discrimination curves for eight subgroups of Cantonese speakers with duration 80 ms.

The scores of the identification and predicted and obtained discrimination for the eight subgroups are plotted in Figures 6–8. Three duration values were selected for illustration: 200, 140, and 80 ms. In general, the musicians showed stronger CP than the non-musicians. For both groups, the perception became more categorical with the increase of the stimulus duration.

To confirm these observations, linear regression models were fitted where correlation values between predicted and obtained discrimination were the dependent variable, and the independent variables included musical experience, vowel quality, duration, and pitch direction. Significant main effects of musical experience [*t*_(46)_ = 2.96, *p* = 0.006], pitch direction [*t*_(46)_ = 2.11, *p* = 0.044], and duration [*t*_(46)_ = 2.46, *p* = 0.013] as well as significant two-way interactions between musical experience and pitch direction [*t*_(46)_ = −3.01, *p* = 0.005] and between musical experience and duration [*t*_(46)_ = −3.20, *p* = 0.003] were obtained. These results indicated that, overall, correlation between predicted and obtained discrimination was stronger for non-musicians than musicians and for the rising pitch direction than the falling pitch direction. In addition, correlation for the rising pitch direction obtained from both musicians and non-musicians was stronger than that obtained for the falling pitch direction obtained from musicians [FM < RM, ${\text{χ}}_{\left(1\right)}^{2}$ = 6.04, *p* = 0.014; FM < RN, ${\text{χ}}_{\left(1\right)}^{2}$ = 5.75, *p* = 0.016] but not from non-musicians [FN < RN, ${\text{χ}}_{\left(1\right)}^{2}$ = 2.35, *p* = 0.125], suggesting that identification better predicted discrimination of the rising pitch contour than the falling pitch contour, particularly among musicians.

Next, we calculated the distances between the predicted and obtained discrimination. A linear regression model suggested a significant two-way interaction between musical experience and vowel quality [*t*_(234)_ = −2.30, *p* = 0.022] and a three-way interaction among musical experience, duration and vowel quality [*t*_(234)_ = −2.09, *p* = 0.021]. *Post-hoc* tests showed that the pairs FN vs. RN [FN > RN, ${\text{χ}}_{\left(1\right)}^{2}$ = 4.146, *p* = 0.042] and MA vs. NI [MA > NI, ${\text{χ}}_{\left(1\right)}^{2}$ = 4.404, *p* = 0.036] were significant. For the three-way interaction, we only found that the distance got larger with the increase of duration, which was true for both groups and for both vowels.

### Summary of the Results

In sum, musicians' perception of pitch direction in a non-native language was generally more categorical than non-musicians. Specifically, they exhibited sharper category boundary and were also more sensitive to between-category differences than non-musicians. In addition, they had greater peakedness of the discrimination functions as well as higher correlation between predicted and obtained discrimination. Surprisingly, however, the relationship between between-category discrimination and duration was significant for non-musicians but not for musicians. In contrast, the relationship between within-category sensitivity and duration was significant for musicians, but not for non-musicians. In general, the musicians had higher scores than the non-musicians. In response to our first research goal, these results strongly suggested that musicians with a tonal language background tended to have stronger CP of pitch in a non-native language.

For our second research goal on examining the effects of stimulus duration, we found that both musicians and non-musicians benefited from increased stimulus duration, but musicians were more sensitive than non-musicians to the changes in stimulus duration reflected by more changes in the sharpness of categoryand the location of category boundary.

Finally, we explored if musicians were more sensitive to factors that may potentially influence pitch processing such as vowel quality and pitch directions. We found that vowel quality did not significantly influence pitch identification among non-musicians, but musicians were more likely to hear more level tones on a high vowel /i/. In addition, both groups showed sharper category boundary for the rising than the falling pitch direction. However, the discrimination results were inconsistent. For between category discrimination, the falling pitch direction had higher *d*-prime scores than the rising pitch direction for musicians, but the reversed pattern was observed for non-musicians. For within-category discrimination, the results showed significant higher d′ scores for the falling pitch than rising pitch.

## Discussion and Conclusion

Using a CP paradigm, this study explored the effects of musical experience, stimulus duration, vowel quality on pitch direction categorization among native Cantonese speakers. Rising and falling pitch direction continua representative of Mandarin Tone 1–2 and Tone 1–4, varying in stimulus duration and vowel height were presented to Cantonese-speaking musicians and non-musicians for identification and discrimination. Sharpness of category derived from the identification data suggested that, in general, musicians exhibited sharper category boundaries than non-musicians, an indication that their perception was more categorical. The results were similar to English-speaking musicians (Chen et al., 2020) and we did not find an obvious ceiling effect of musical experiences reported for the Mandarin-speaking musicians in their study. In addition, albeit statistically non-significance, category boundaries were sharper for the rising pitch direction than the falling pitch direction, and for the high vowel [i] than the low vowel [a] for both groups of listeners. These results suggested the universal psychoacoustic effects of pitch direction and vowel intrinsic *F*_0_, but a unique effect of musical experience on pitch direction identification. Specifically, rising *F*_0_ and higher intrinsic *F*_0_ associated with a high vowel led to more precise perceptual boundaries between a level and a rising pitch contour among both groups of listeners. However, greater auditory sensitivity to both *F*_0_ parameters (i.e., rising *F*_0_ and higher intrinsic *F*_0_) among musicians than non-musicians resulted in their significantly more abrupt shift in category boundaries.

In addition to sharper identification category boundary, discrimination patterns also suggested that musicians' perception of pitch direction was more categorical than that of non-musicians. Specifically, averaged across vowel height, pitch direction and duration, musicians were more sensitive to between-category differences than non-musicians. Peakedness of the discrimination function also suggested that musicians' perception of pitch direction was more categorical than that of non-musicians. That is, on average, peakedness values were larger for musicians than for the non-musicians, and for the rising than the falling pitch direction. There was also a general trend of greater peakedness with the increase of duration for both groups of participants. Moreover, although musicians had larger peakedness than non-musicians for the falling pitch direction, the two groups had comparable peakedness for the rising pitch direction. Correlation between predicted and obtained discrimination also pointed to a stronger CP among musicians as it was found that the correlation was stronger for non-musicians than musicians and for the rising pitch direction than the falling pitch direction, particularly among musicians. Regarding distances between predicted and obtained discrimination, they were numerically greater among musicians than non-musicians, but the differences did not reach statistical significance. In addition, the distance became larger as the stimulus duration increases for both groups. The effect of pitch direction was observed only among non-musicians, with greater distance for falling than rising pitch direction. In sum, against characteristics of CP, musicians' perception of pitch direction was generally more categorical than non-musicians. They exhibited sharper category boundary, greater between-category sensitivity, greater peakedness of the discrimination functions, higher correlation between predicted and obtained and greater distance between predicted and obtained discrimination.

In addition to the rising tone being inherently more salient, the possible effects of pitch range cannot be ruled out. As pointed out by an anonymous reviewer, the two Mandarin tonal pairs (T1 vs. T2, T1 vs. T4) are different in the pitch range. According to the traditional five-point scale, the tonal contour changes from point 3 to point 5 for the T2[35]/T1[55] pair, but it changes from point 5 to point 1 for the T1[55]/T4[51] pair. To control for potential confounding effects of differences in acoustic distance between members in both set of stimuli in a CP paradigm, same acoustic differences among stimuli in both tone continua are necessary. However, although the tonal continua are not natural Mandarin tonal continua, a smaller amount of *F*_0_ fall for some members of the falling continuum (e.g., [12], [13]), may render them less “natural” and less acoustically salient than their rising counterparts in the rising continuum. This, in turn, may lead to better discrimination for the rising continuum. However, our discrimination results suggest that this might not always have been the case. For between-category discrimination, the falling pitch direction had higher *d*-prime scores than the rising pitch direction for musicians, but the reversed pattern was observed for non-musicians. For within-category discrimination, the results showed significant higher d′ scores for the falling pitch than rising pitch. These results suggest that musicality has a stronger effect on discrimination pattern than pitch range.

Our findings suggest that musicians may be at an advantage in learning tones of a second language in that they may better categorize tones in a new tonal language. Cantonese has three level tones and two rising tones, which are different from Mandarin tonal categories (one level tone and one rising tone). Although there might be some positive transfer from Cantonese tones, learners need to establish or revise the existing categories. The beneficial effects from musical training were more obvious for musicians of non-tonal speakers processing tones (Peng et al., 2010; Zhao and Kuhl, 2015a; Chen et al., 2020) than tonal speakers. Our results were inconsistent with what have been found for tone processing in the native language by musicians (Wu et al., 2015; Chen et al., 2020), suggesting that musical training may help tone processing more in a non-native tone language. It has been argued that musical training help improve auditory memory (Patel, 2011), representations of auditory objects and the mapping between new stimuli and existing memory templates (Bidelman, 2017). In addition, the sensitivity to linguistic and music pitch processing may come from a general cognitive processing (Perrachione et al., 2013). However, musical measures that were indirectly related to tones were less likely to predict tonal word learning (Bowles et al., 2016).

Furthermore, musicians were also more sensitive than non-musicians to changes in stimulus duration when identifying pitch directions. Both musicians and non-musicians benefited from increased stimulus duration, but musicians were more sensitive than non-musicians to the changes in stimulus duration reflected by greater changes in the sharpness of category. In addition, category boundary locations calculated from the identification functions showed that category boundary had a smaller value as the stimulus duration increased for both groups of listeners and musicians had a smaller value than non-musicians. Similar to the findings in Chen et al. (2020), musicians may benefit more from the extra time in that they can use the time to form a more robust auditory representation and matching sounds to internalized memory templates. Since musicians in our study are also native Cantonese speakers, they can rely on existing long-term memory of Cantonese lexical level and rising tonal categories in processing stimuli and further benefit from context-coding, matching sounds to long-term representations.

Both groups were also affected by intrinsic *F*_0_s associated with vowel height, but only musicians appeared to be perceptually compensated for the effects. Compared to those with lower musical capacity, listeners with higher musical capacity were shown to be more capable of teasing apart acoustic cues and employ more of *F*_0_, which is a key acoustic cue in pitch processing (Cui and Kuang, 2019). On the other hand, those with lower musical ability tend to rely on both spectral and *F*_0_ cues. Our results thus showed that musical training affords musicians the ability to better accommodate variation in *F*_0_ induced by factors.

While both groups showed similar effects of pitch direction in identification (i.e., sharper category boundary for the rising than the falling pitch direction), the effects of the two pitch directions were inconsistent in discrimination. The identification results were consistent with the reported difficulties that Cantonese speakers have in differentiating Mandarin Tone 1 (high-level) from Mandarin Tone 4 (high-falling) (Hao, 2012). With three contrastive level tones of different *F*_0_ height, Cantonese speakers pay more attention to *F*_0_ height than *F*_0_ contour in comparison to Mandarin speakers (Peng et al., 2012). Also, although Cantonese has two rising tones (Tone 2 [25] and Tone 5 [23]), it only has a falling tone with a shallow slope (Tone 4 [21]), which may lead to their difficulties in identifying falling tones.

Though the factor of proficiency of Mandarin has been controlled between musicians and non-musicians, their exposure to Mandarin may have an effect on CP of their Mandarin lexical tones. Note that non-tonal learners of a tonal language may establish tonal categories and perceive tones in a more categorical way (Shen and Froud, 2016). However, Cantonese learners of Mandarin usually have difficulties in perceiving Mandarin tones. Cantonese speakers pay more attention to *F*_0_ height than *F*_0_ contours compared to Mandarin speakers due to three contrastive Cantonese level tones of different *F*_0_ height in their tonal system (Peng et al., 2012). Also, Cantonese has two rising tones (Tone 2 [25] and Tone 5 [23]), but only a falling tone with a shallow slope (Tone 4 [21]), leading to their difficulties in identifying falling tones. Despite the learning experience of Mandarin, the identification results showed difficulties of Cantonese learners in differentiating Mandarin Tone 1 (high-level) from Mandarin Tone 4 (high-falling) (Hao, 2012). Since Cantonese has both level and rising tones, positive transfer may occur when they perceive Mandarin tones. Our data showed Cantonese musicians had sharper categorical boundary in perceiving rising tones than falling tones, though the differences were less discernible in non-musicians.

Moreover, variability in musical ability and L2 proficiency may influence tone processing. Li and DeKeyser (2017) reported that musical tonal ability is positively correlated with the accuracy rate of perception of tones and comprehension of tone words after training. Also, it has been reported that those with higher musical ability tend to separate acoustic cues better. *F*_0_ is known as a cue most relevant to pitch processing, and listeners with higher musical ability could tease apart spectral cues and *F*_0_ and rely on *F*_0_ only, which may help in tone processing (Cui and Kuang, 2019). Wong and Perrachione (2007) reported that individuals' learning results were positively correlated with their musical experience. Those who received more private music lessons performed better in a Mandarin tone learning task. In addition, it has been reported that prior musical experience and musical aptitude scores predicted success in learning tonal word (Cooper and Wang, 2012) and musical aptitude scores were also positively related to tone discrimination ability (Delogu et al., 2006, 2010). In future studies, it is worth exploring how variability in musical ability and CP are related and whether more musical experience and higher capability will lead to greater sensitivity to vowel quality and more benefits from longer stimulus duration.

The variability in L2 proficiency may also influence tone processing though the results have not been consistent. It has been reported that fluent tone language speakers performed significantly better than less fluent tone language speakers and non-tone language speakers (Deutsch et al., 2009). Hao (2018) tested how proficiency level affects tone discrimination for native English speakers. Three groups of speakers were recruited, including beginning, advanced learners and those who were naïve to Mandarin. Higher accuracy were obtained by learners than those speakers naïve to Mandarin. However, both group of learners were accurate in discriminating tonal pairs except for the T2–T3 pair without significant differences. With extensive exposure to a tone language, adult listeners of non-lexical tone languages may exhibit categorical-like perception for non-native lexical tones. For example, Shen and Froud (2016) reported that native speakers of American English who are advanced learners of Mandarin perceived Mandarin tonal continua in a categorical-like manner, evidencing sharp category boundaries and prominent discrimination peaks. In fact, their discrimination performance was found to be better than that of native Mandarin listeners who had been living as university students in the US for a few years. These results suggest that CP of non-native phonetic categories can be acquired through intensive and long-term exposure. According to listeners' reports in this study, the participants were proficient non-native Mandarin speakers. It is expected that Cantonese speakers with lower proficiency or naïve to Mandarin may show weaker CP. Therefore, whether musical training may contribute more to tone processing in the initial stage of learning a tone language is worth exploring in future studies.

## Data Availability Statement

The raw data supporting the conclusions of this article will be made available by the authors, without undue reservation.

## Ethics Statement

The studies involving human participants were reviewed and approved by Human Subject Ethics Sub-committee, the Hong Kong Polytechnic University. The patients/participants provided their written informed consent to participate in this study.

## Author Contributions

SC and RW contributed to the conception and design of the study. YY collected data performed the statistical analysis based on code written by SC. SC, YY, and RW wrote and revised the manuscript. All authors contributed to the article and approved the submitted version.

## Conflict of Interest

The authors declare that the research was conducted in the absence of any commercial or financial relationships that could be construed as a potential conflict of interest.

## Publisher's Note

All claims expressed in this article are solely those of the authors and do not necessarily represent those of their affiliated organizations, or those of the publisher, the editors and the reviewers. Any product that may be evaluated in this article, or claim that may be made by its manufacturer, is not guaranteed or endorsed by the publisher.

## Footnotes

^1^Following the Tukey's method, outliers are defined as values above and below the 1.5 * IQR (interquartile). The script for detecting and removing the outliers can be found at: https://goo.gl/4mthoF.
